# A Review of Damage Tolerance and Mechanical Behavior of Interlayer Hybrid Fiber Composites for Wind Turbine Blades

**DOI:** 10.3390/ma18102214

**Published:** 2025-05-10

**Authors:** Amir Baharvand, Julie J. E. Teuwen, Amrit Shankar Verma

**Affiliations:** 1 Department of Mechanical Engineering, University of Maine, 75 Long Road, Orono, ME 04469, USA; amir.baharvand@maine.edu; 2 Advanced Structures and Composites Center, University of Maine, 35 Flagstaff Road, Orono, ME 04469, USA; 3 Faculty of Aerospace Engineering, Delft University of Technology, Kluyverweg 1, 2629 HS Delft, The Netherlands; j.j.e.teuwen@tudelft.nl

**Keywords:** damage tolerance, fiber hybridization, low-velocity impact, mechanical properties, wind turbine blade, composite structures

## Abstract

This review investigates interlayer hybrid fiber composites for wind turbine blades (WTBs), focusing on their potential to enhance blade damage tolerance and maintain structural integrity. The objectives of this review are: (I) to assess the effect of different hybrid lay-up configurations on the damage tolerance and failure analysis of interlayer hybrid fiber composites and (II) to identify potential fiber combinations for WTBs to supplement or replace existing glass fibers. Our method involves comprehensive qualitative and quantitative analyses of the existing literature. Qualitatively, we assess the damage tolerance—with an emphasis on impact load—and failure analysis under blades operational load of six distinct hybrid lay-up configurations. Quantitatively, we compare tensile and flexural properties—essential for WTBs structural integrity—of hybrid and glass composites. The qualitative review reveals that placing high elongation (HE)-low stiffness (LS) fibers, e.g., glass, on the impacted side reduces damage size and improves residual properties of hybrid composites. Placing low elongation (LE)-high stiffness (HS) fibers, e.g., carbon, in middle layers, protects them during impact load and equips hybrid composites with mechanisms that delay failure under various load conditions. A sandwich lay-up with HE-LS fibers on the outermost and LE-HS fibers in the innermost layers provides the best balance between structural integrity and post-impact residual properties. This lay-up benefits from synergistic effects, including fiber bridging, enhanced buckling resistance, and the mitigation of LE-HS fiber breakage. Quantitatively, hybrid synthetic/natural composites demonstrate nearly a twofold improvement in mechanical properties compared to natural fiber composites. Negligible enhancement (typically 10%) is observed for hybrid synthetic/synthetic composites relative to synthetic fiber composites. Additionally, glass/carbon, glass/flax, and carbon/flax composites are potential alternatives to present glass laminates in WTBs. This review is novel as it is the first attempt to identify suitable interlayer hybrid fiber composites for WTBs.

## 1. Introduction

### 1.1. Background: Impact Loads on Wind Turbine Blades and Design Approaches

Several countries are focusing on expanding their wind energy capacities such as the US plans to install 30 gigawatts (GW) of offshore wind energy by 2030 [1]. Modern offshore wind turbines (OWTs) can have power outputs of 12–15 megawatts (MW) and rotor diameters up to 236 m [2,3,4,5]. Scaling up wind turbines brings challenges, including increasing aerodynamic and gravity loads on wind turbine blades (WTB) and complexities in handling these components during transportation and assembly [6,7,8].

WTBs are conventionally constructed using sandwich structures that typically incorporate balsa or foam cores and glass fiber-reinforced composites. The structural design methodology of these blades is largely based on a safe-life design approach where partial safety factors are employed to ensure both the static strength (or load-carrying capacity) and the fatigue service life of the blades meet requisite standards [9,10]. Partial safety factors are critical in this context as they help mitigate the uncertainties arising from various sources, including assumptions made during numerical analysis and variability inherent in manufacturing processes. A recent report by Det Norske Veritas (DNV) [11] indicates that blade designers tend to utilize lower partial safety factors and push the reserve margin to zero (refer to Appendix A for the definition of some of the key terms used in this review article). However, a small reserve margin could trigger different failure mechanisms, e.g., delamination and buckling, if the manufacturing defects and unexpected events such as impact loads are not explicitly considered during the blade design [12].

**Figure 1:**
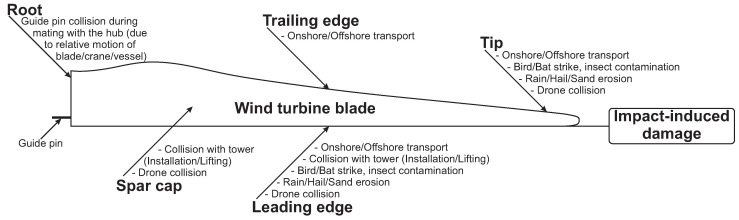
The fishbone diagram of impact loads and impact-susceptible regions in a wind turbine blade [12].

**Figure 2:**
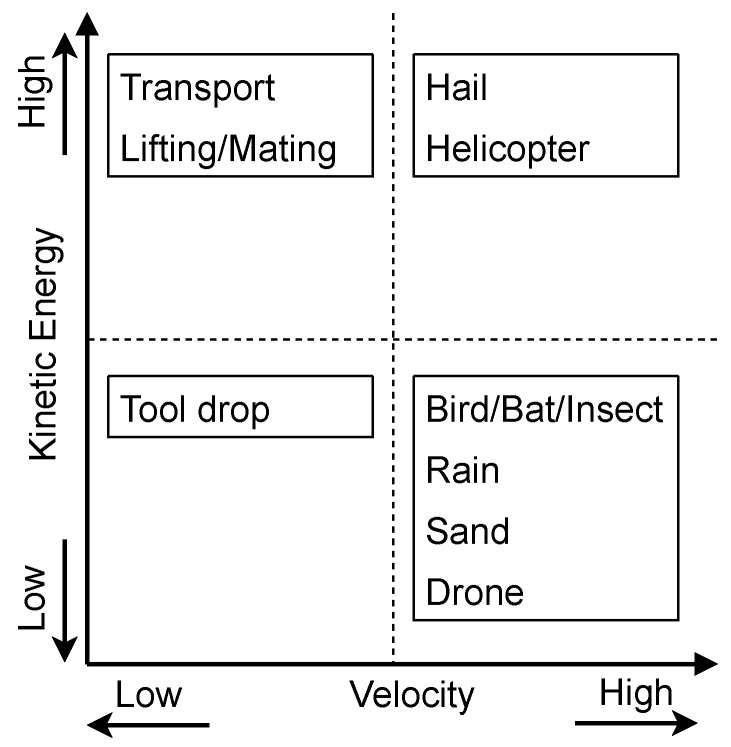
An overview of the kinetic energy and velocity of different accidental and operational impact loads in WTBs (data obtained from [12]).

Transverse impact loads, often overlooked in the design phase, are a significant source of damage to WTBs during transport, service, and maintenance [12,13]. Sources of impact loads on WTBs are wildlife (bird/bat strike), hydrometeors (hailstone and rain erosion), airborne particles (sand erosion and insect contamination), and accidental impact loads during operation and maintenance [12]. A review of different sources of impact loads on WTBs is provided in [12] and is also summarised in Figure 1. Furthermore, Figure 2 provides a qualitative categorization of these impact loads based on their velocity and kinetic energy. This classification reveals that WTBs are subjected to a range of impact loads, varying in both velocity and energy.

Studies [14] have shown that composite laminates can suffer reductions in compressive and flexural strength by up to 20% and 70%, respectively, after low-velocity impacts (LVI). Therefore, incorporating considerations for impact loads is vital in blade design. However, if WTBs are to be designed using the current safe-life design approach to withstand impact loads, it would necessitate the use of large safety factors and reserve margins to compensate for impact-induced damages. This is expected to result in heavy, inefficient, and expensive blades. Consequently, there is a growing necessity to shift from the safe-life design to a more damage-tolerant design methodology that allows for damages and lower safety factors while maintaining the blade’s structural integrity [9,15,16].

Damage tolerance design (DTD) assumes the existence of inherent flaws and induced damages in the structure. Specifically, DTD identifies regions of the highest stress within the structure in relation to the applied load as the most susceptible to damage [17]. Further, the structure is required to retain its load-carrying capacity in the presence of damage and during damage growth while maintaining a minimum level of residual static strength for an assumed service period [18]. DTD encompasses the concept of damage growth and residual properties to achieve structural integrity (I) through damage-tolerant materials and damage retaliation mechanisms (i.e., crack-arresting) and/or (II) through lower safety factors, design load redistribution, use of sensors for structural health monitoring, and scheduled inspection [19]. This review focuses on fiber hybridization, which is considered a promising method to improve the damage tolerance of WTBs [16].

### 1.2. Fiber Hybridization

In the DTD framework, material selection plays a pivotal role in ensuring a structure’s capability to sustain loads despite the presence of damage or inherent flaws [16]. Within this context, fibers and resin are critical elements as far as the DTD of WTBs is concerned. This review focuses on fibers, given their role as the primary load-bearing component in fiber-reinforced composites, and their significant influence on crack initiation within the resin matrix due to interactive effects [20].

In conventional WTBs, synthetic fibers, such as E-glass and carbon, are predominantly employed to meet the requirements for strength, stiffness, and fatigue resistance. Conversely, natural fibers have been found to have an application mainly in smaller WTBs [21]. An in-depth analysis of the latest materials and methods devised to augment the damage tolerance of WTBs is thoroughly examined in [16]. Among these methods, fiber hybridization stands out as a technique for improving impact resistance and damage tolerance in composites. This approach involves integrating two or more types of fibers within a resin matrix, resulting in what is termed a hybrid fiber composite (or hybrid composite). The process of combining different fibers to create these hybrid composites is known as fiber hybridization, a method that has been explored and refined in numerous studies [22,23,24,25,26,27,28,29,30].

In hybrid composites, the integration strategy typically involves substituting a portion of low elongation (LE) and high stiffness (HS) fibers, which are primarily responsible for load-bearing, with high elongation (HE) and low stiffness (LS) fibers [31]. This strategic substitution results in the attainment of superior mechanical properties and improved impact behavior, surpassing the expected performance of either individual non-hybrid LE-HS or HE-LS composites [32]. This enhanced performance is attributed to the hybrid or synergistic effect, stemming from the combined presence of LE-HS and HE-LS fibers, their interaction at the interface, and their bonding with the resin [33]. To elucidate the distinction between LE-HS and HE-LS fibers, Table 1 lists the stiffness (tensile modulus in the fiber direction) and percent elongation at the break of several common fibers. These mechanical properties illustrate the classification of LE-HS and HE-LS fibers within a hybrid composite. For example, in a hybrid E-glass/carbon composite, E-glass—with relatively lower stiffness and higher elongation—is categorized as HE-LS fiber, while carbon—with relatively higher stiffness and lower elongation—is categorized as LE-HS fiber.

**Figure 3:**
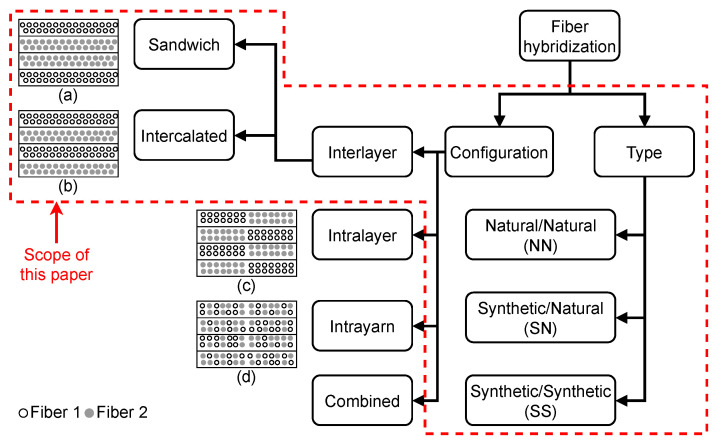
The breakdown of fiber hybridization. A schematic of a hybrid composite with four layers is provided next to each hybrid fiber configuration: (**a**) Interlayer (sandwich), (**b**) interlayer (intercalated), (**c**) intralayer, and (**d**) intrayarn.

Hybrid fiber composites can be manufactured by hybridizing natural/natural (NN), synthetic/natural (SN), and synthetic/synthetic (SS) fibers and categorized into four configurations, as demonstrated in Figure 3: (I) interlayer, where layers of different fibers are stacked on each other during manufacturing (e.g., during fiber deposition process of a vacuum infusion), (II) intralayer, where distinct fiber yarns are woven together or placed next to each other in bundles, (III) intrayarn, where a fiber bundle is formed by mixing different fibers, and (IV) combinations of configurations (I)–(III) [32]. Most research focuses on interlayer and intralayer configurations [32], with the interlayer configuration often reporting higher mechanical properties under certain load conditions, like compressive and tensile stresses [33,35]. This is due to the high dispersion degree of distinct fibers, which could lead to uneven stress distribution and negatively affect the strength and failure strain of the intralayer hybrid composites [33,36]. Given its relative simplicity and cost-effectiveness in manufacturing, as mentioned by [32], this review focuses on interlayer hybrid composites. In these composites, fibers in the form of fabric are arranged in specific lay-up configurations, such as the sandwich or intercalated lay-ups. The sandwich lay-up configuration involves placing layers of the same fiber type on either side of a core made of a different fiber (see Figure 3a), while the intercalated lay-up configuration refers to the alternating layers of different fibers (Figure 3b).

Although this review primarily focuses on interlayer hybrid composites, it is important to note that numerous factors, including fabrication method, fiber dispersion, and fiber volume fraction (FVF), affect the mechanical behavior of hybrid composites. Interlayer hybrid composites are often easier to fabricate using conventional lay-up techniques such as hand lay-up and vacuum infusion. However, they offer less control over fiber dispersion and layer thickness than intralayer and intrayarn configurations, which require specialized equipment to achieve uniform and tailored properties [37]. FVF plays a critical role in tailoring synergistic and hybrid effects. Increasing the content of LE-HS fibers, e.g., carbon, generally enhances their tensile and flexural properties [38]. Conversely, increasing HE-LS fiber content, e.g., glass, improves the failure strain and leads to a pseudo-ductile behavior. The pseudo-ductile behavior is characterized by a progressive, non-catastrophic stress-strain response that delays failure [39,40,41,42]. This behavior can be achieved through a specific interlayer hybrid lay-up configuration, where a thin layer of LE-HS fibers is placed between two thick layers of HE-LS fibers [41]. However, studies have shown that thick layers of HE-LS fibers on the impacted side could lead to larger delamination sizes at the interface of distinct fibers [43,44]. On the other hand, increasing LE-HS fiber content results in permanent damage and reduces post-impact residual compressive strength, as most of the impact energy is absorbed through the fiber breakage of these fibers [45]. The fiber content and dispersion also affect the fatigue behavior of hybrid composites. Intrayarn hybrid rods with uniformly dispersed LE-HS and HE-LS fibers—due to better interfacial bonding—exhibit enhanced fatigue behavior compared to other hybrid configurations [46]. Furthermore, the fatigue behavior at different stress levels may shift depending on the specific type of fiber. Carbon fibers dominate at high-stress levels, while glass fibers govern the fatigue behavior at low-stress levels [47]. Overall, the mechanical behavior of hybrid composites depends on various interacting factors, and it is essential to recognize these interactions when designing hybrid composites.

### 1.3. Scope, Novelty, and Structure of the Review Paper

The present review paper has two main objectives: (I) to assess the effect of different hybrid lay-up configurations on the damage tolerance of interlayer hybrid fiber composites with emphasis on impact loads, and (II) to identify potential fiber combinations for wind turbine blades that can supplement or replace existing glass fibers. Recently, with increased blade length and weight, recyclability issues, and carbon footprint of glass fibers [48], hybrid composites have become one of the important research areas for future WTBs [32,37,49,50,51,52]. The strategic choice of fibers and their placement within a hybrid lay-up could lead to cost-effective lighter blades with improved stiffness compared to the conventional WTBs made of E-glass [53]. Additionally, interlayer fiber hybridization does not change the existing manufacturing process of WTBs, as the same layering techniques with the E-glass fibers can be used to manufacture WTBs made of hybrid composites [54].

Fiber hybridization is a broad topic. There has been an extensive literature review published on interlayer hybrid composites [25,31,32,55,56]. These include reviewing mechanical properties under various load conditions, developing mechanical models, manufacturing methods, and the benefits of hybrid composites in enhancing damage tolerance and their structural applications. Factors, including ratios of fibers involved in hybridization, hybrid lay-up configuration, and fiber preparation process, influence the mechanical properties, fatigue properties, and durability of hybrid composites. However, the effect of hybrid lay-up on the failure analysis under various load conditions and the damage tolerance of interlayer hybrid composites has not received much attention. Also, no systematic attempt has been made to compare the mechanical properties of different interlayer hybrid composites. Therefore, this review aims to fill these gaps by analyzing the influence of hybrid lay-up configurations on damage tolerance and failure analysis of hybrid composites and recognizing alternative fiber combinations to glass for WTBs.

The novelties of the present review are: (I) a comprehensive assessment of the underlying failure modes and damage tolerance of various hybrid lay-up configurations using an aerospace-inspired DTD framework with a specific focus on the effects of impact loads on WTBs, (II) a simultaneous comparison among the fundamental mechanical properties (tensile and flexural) of hybrid and non-hybrid composites, and (III) recognizing potential fibers for hybridization for WTB application through a systematic quantitative analysis. The scope of this review (Figure 3) is limited to interlayer hybrid composites with the sandwich and intercalated lay-up configurations. Throughout the text, hybrid composite refers to interlayer hybrid composite.

**Figure 4:**
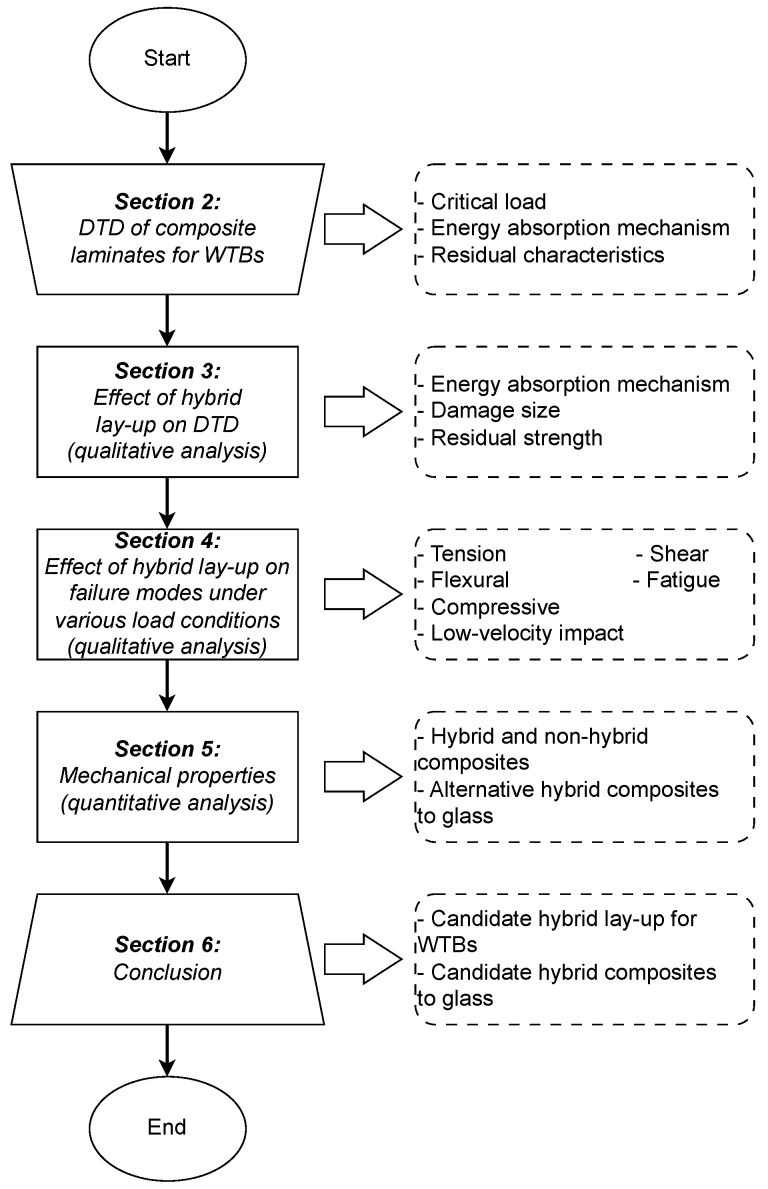
An overview of the methodology used in this review.

The structure of the present review is illustrated in Figure 4 and outlined as follows. First, we present the key elements of DTD for composite laminates in WTBs in Section 2, where an aerospace-inspired DTD framework is introduced. This would form the foundation for our discussions in the rest of the document, especially regarding impact loads (referenced in Section 2). Next, a qualitative assessment of damage tolerance and failure modes of different interlayer hybrid lay-up configurations are addressed in Section 3 and Section 4, respectively. A quantitative analysis of the mechanical properties of hybrid and non-hybrid composites, the hybrid effects, and alternative hybrid composites to the glass composite are presented in Section 5. Finally, concluding remarks and recommendations for future research are given in Section 6 and Section 7.

### 1.4. Study Limitations

Although the findings of this study can be applied to any composite structures, our analysis addresses load conditions unique to WTBs throughout their service life. While most regions of WTBs are sandwich structures with balsa or foam cores and composite skins, this paper focuses explicitly on hybrid composite laminates, essential for load-carrying components such as spar caps critical to the blade’s structural integrity. Furthermore, the surveyed literature is limited to LVI (less than 10 m/s) and includes the discussions from Izod, Charpy, and drop-weight impact tests. Therefore, the discussion made in the paper covers non-repeated hard-body impact with a metallic projectile and does not include impact events such as bird and bat strikes. Furthermore, the insights and conclusions drawn from mechanical tests—such as tensile, flexural, and impact tests—are based on studies conducted at the coupon scale. Differences in size, boundary conditions, projectile and target geometry, impact angle, stacking sequence, fiber architecture, and resin types challenge the direct extrapolation of our findings to larger structures of typical WTBs. As such, while our insights provide a valuable understanding of hybrid composites, the scale-up from coupon level to full-scale blades requires tailored-modeling techniques and comprehensive empirical validation. Additionally, modeling [32,39,40,57,58,59] and manufacturing techniques [54,60,61,62], sustainability of fibers and resin, and environmental effects [26,27,60,63,64,65,66]—while significant in the operational life of composite structures—are beyond the scope of this review. For more in-depth information, readers can refer to the references provided above.

## 2. DTD of Composite Laminates for WTBs

### 2.1. Key Elements in DTD of Composite Laminates Based on Aircraft Structures

**Figure 5:**
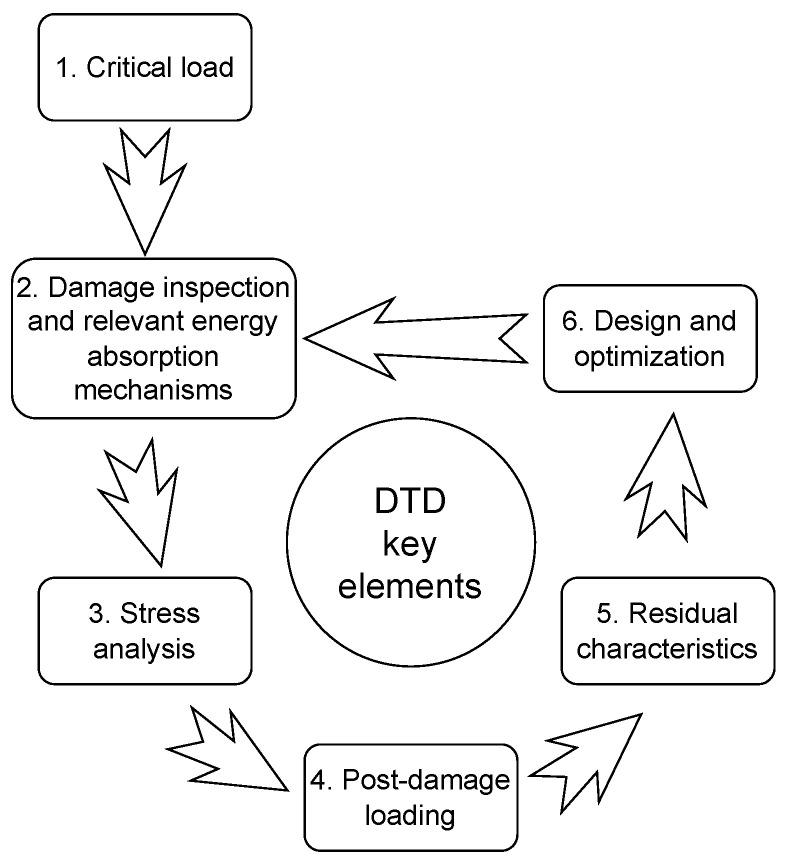
Key elements of the DTD framework (adapted from [17]).

The field of WTBs has witnessed extensive research on damage tolerance, as highlighted in [9,15,16,67]. However, there remains a notable gap in the formulation of specific requirements for the DTD of composite laminates for WTBs. Addressing this gap, this section focuses on discussing the key elements in DTD for composite laminates for WTBs, drawing principles from the established DTD frameworks for composite laminates in aircraft structures, as detailed in [17,68]. In this review, we have adopted this framework for discussing the DTD of interlayer hybrid composites in the context of WTBs, with a particular emphasis on impact loads. According to the principles laid out by [17] (see Figure 5), the key elements in DTD of composite laminates encompass:

1. *Critical Load:* The DTD framework begins with the identification of the most critical load expected to affect a structure during its service life, specifically focusing on loads that could lead to unacceptable structural damages. This involves evaluating all potential load scenarios and determining which poses the greatest risk to the structure’s integrity.
2. *Damage Inspection and Relevant Energy Absorption Mechanisms (EAM):* This aspect entails inspecting and identifying the EAMs associated with the critical load. It involves a detailed quantification of the size, location, and distribution of damages, which are crucial for understanding the effect of the critical load on the structure.
3. *Stress Analysis:* A comprehensive stress analysis is conducted in the vicinity of the damage. This analysis aims to ascertain the level of criticality of the damage, determining whether it is likely to undergo stable or unstable growth, which is vital for planning appropriate responses.
4. *Post-Damage Loading:* This element is concerned with characterizing the progression of damage in relation to cyclic loads, such as gravity and aerodynamic loads. It examines the relationship between damage growth and the number of cycles, considering different cyclic load directions, for example, tension-compression cyclic load.
5. *Residual Characteristics:* Assessing the post-damage structural capacity is key in this aspect. It involves measuring the quasi-static properties (like compressive strength and modulus) and determining the fatigue life, providing insights into the structure’s performance after sustaining damage.
6. *Design and Optimization:* The final element involves employing various strategies to enhance the damage tolerance of the structure. This could include modifying the stacking sequence or other design parameters to improve the overall resilience of the structure to the identified critical loads.

Based on the abovementioned key elements, the rest of this section discusses how this aerospace-inspired framework can be applied to the DTD of composite laminates for WTBs with emphasis on impact loads. The discussion is limited to only three critical elements: critical load (key element 1), EAM (key element 2), and the relevant residual characteristics (key element 5). The discussion on stress analysis (key element 3), post-damage loading (key element 4), design and optimization (key element 6), and damage inspection (part of key element 2) is considered out of the scope of this paper. A summary of the key elements identified for DTD framework for composite laminates for WTBs is provided in Table 2 and also discussed below.

### 2.2. Critical Load

Transverse impact load is considered the critical loading in this review paper for WTBs. Impact, particularly LVI, is often associated with internal damage, such as matrix cracking and delamination, without clear surface indicators, a condition referred to as barely visible impact damage (BVID) [69,70]. BVID is difficult to detect and is often regarded as a threat to the long-term structural integrity of composite structures [71]. Notably, impact-induced delamination, although initially stable, can act as a crack precursor that grows under operational and environmental conditions, particularly under compressive loads, leading to buckling, a common cause of catastrophic failure of WTBs [72]. Additionally, even minor impact energies (less than 10 J) can result in a notable loss of compressive strength in composite laminates, especially when delamination is present [73].

### 2.3. Energy Absorption Mechanisms and Damage Size

Matrix cracking, fiber breakage, and delamination are identified as the dominant EAMs during LVI in WTBs [74,75]. Matrix cracking refers to cracks within the polymeric matrix, typically occurring at the early stages of an impact event due to highly localized contact stress at the impacted zone in thick laminates and bending stress at the laminate back face in thin laminates [76,77,78,79]. These cracks may extend along the fiber direction during in-service loading [70]. Although matrix cracking alone absorbs relatively low energy during an impact event and may negligibly affect the load-carrying capacity of a composite laminate [80,81], it often serves as a precursor to more critical EAMs, i.e., delamination [76]. Fiber breakage, on the other hand, occurs at relatively high impact energies [70], where compressive stress at the impacted zone and bending stress at the laminate back face are pronounced [76]. Delamination—the separation of adjacent layers in a composite laminate—is often considered the critical EAM [71]. Delamination is caused by interlaminar shear stress resulting from impact forces and transverse shear, as well as tensile cracks at the laminate back face, which is particularly important in thin laminates due to the high bending stress [76,82]. While all three damage mechanisms contribute to energy absorption under impact loads, delamination is widely recognized as the dominant EAM [71]. Delamination poses a high risk to the structural integrity of composite structures due to its classification as BVID [68,83]. Furthermore, the literature suggests that the damage size and residual properties in composite laminates are more influenced by delamination than matrix cracking [79,84]. Delamination can create weakened areas that are interconnected and difficult to detect [17,68,70,85]. Consequently, delamination is considered the key EAM influencing damage size in this review. For the purposes of this review, damage size is referred to as delamination size, which is defined as the area in the composite induced by delamination during LVI (see Table 2).

### 2.4. Residual Characteristics

In the DTD of composite laminates for WTBs, the assessment of residual characteristics is vital. These characteristics reveal the laminate’s load-bearing capacity after impact. According to [86], evaluating these properties in the context of inter-fiber failure, such as fiber/matrix debonding, is essential. However, in DTD, the focus is on residual strength as influenced by the most significant impact damage, namely delamination [17,71].

#### 2.4.1. Relationship Between Residual Strength and Damage Size

**Figure 6:**
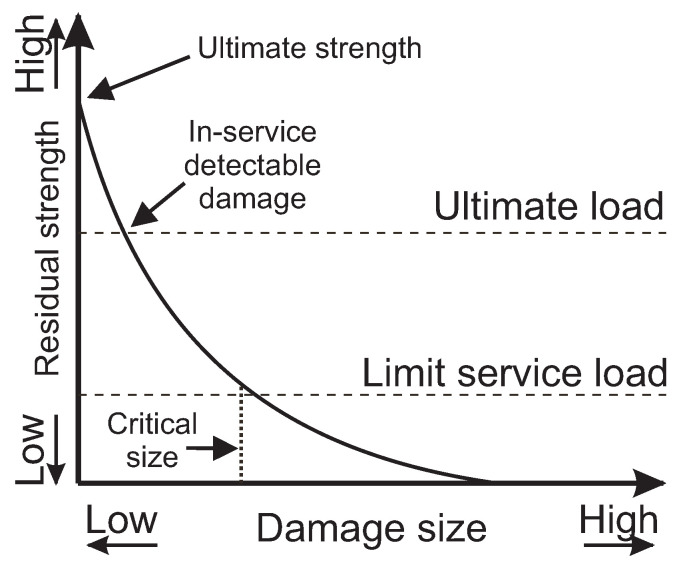
The relation between damage size and residual strength [17].

Understanding the relationship between residual strength and damage size is pivotal in the evaluation of DTD for composite laminates. Figure 6 elucidates how residual strength declines as damage size increases. This critical relationship is characterized by four distinct points, each corresponding to a specific load condition considered in design assessments:

1. **Ultimate strength**—This is the highest residual strength a pristine composite can exhibit, signifying its capability to bear the maximum load.
2. **Ultimate load**—This is the residual strength corresponding to the ‘design load’ as defined in [86], which the composite should maintain despite these damages being undetected, such as porosity or minor delaminations.
3. **Limit service load**—This is the minimum residual strength that a damaged composite should uphold until repair to guarantee operational safety and structural integrity. This also corresponds to the ‘characteristic load’ in [86].
4. **Critical size**—The point at which the residual strength falls below the limit service load due to damage, necessitating immediate repair.

Non-destructive testing (NDT) is often used to characterize the damage size during service. While numerous techniques exist, we highlight several commonly used techniques for in-service characterization of delamination size in composite laminates. The accurate determination of delamination size depends on the physical interaction between the damage and the probing energy—ultrasonic, thermal, optical, or acoustic—used in a specific NDT technique [87]. Ultrasonic C-scan is one of the most widely used techniques for detecting and determining the planar size and depth of delamination. It uses high-frequency sound waves—typically in the range of 0.5–10 MHz [88]—through the composite laminate to detect discontinuities based on the reflection or transmission of the sound waves [87,89]. Infrared thermography is another useful technique for determining the size of delamination in composite laminates. This technique uses variations in surface temperature to detect the location and delamination size [87,89]. However, its accuracy decreases for deeper delamination due to limited heat fluctuations [90]. Shearography is another effective technique for determining the delamination size. This technique uses subsequent imaging of the deformed composite laminate using an illuminated laser beam to create a fringe pattern, or the so-called shearogram, which enables the detection and determination of delamination size [89]. Acoustic emission is another technique for monitoring the damage initiation and propagation during in-service loading. It is based on the sudden release of energy of sound waves and can be used to detect matrix cracking, fiber breakage, and delamination [91]. However, this technique is incapable of quantifying the delamination size and depth, and it offers qualitative rather than quantitative metrics, i.e., higher released energies are related to fiber breakage. Additionally, this technique requires further post-processing to identify specific EAMs [87]. Overall, the choice of the NDT techniques depends on the required resolution, inspection depth, and accessibility of the delamination [92]. Nevertheless, NDT techniques are essential for quantifying delamination size and correlating it with the residual strength of composite laminates.

#### 2.4.2. Key Residual Mechanical Properties in WTBs Affected by Delamination

This section explores the correlation between residual strength and delamination size, aiming to identify the key residual mechanical properties in WTBs affected by delamination.

*Tensile strength:* Delamination has a minor effect on the in-plane tensile strength of composites as the fibers close to the delamination zone can retain their load-carrying capacity under tension [71,73,93,94,95,96]. For instance, the tensile strength of the spar cap on the pressure side of a WTB shows only an 11% decrease in the presence of delamination [97]. Therefore, this mechanical property due to delamination in WTBs is not considered in this review paper.
*Flexural strength:* Delaminations induced by shear stress at the laminate mid-section can affect the flexural strength of composite laminates by changing shear stress distribution [98]. Also, during the flexural load, delaminations on the compressive side of the laminate, i.e., the suction side of the spar cap, can reduce the flexural strength because of induced local buckling [95,99].
*Compressive strength:* Typically, delamination influences the compressive strength of the composite laminates. The reduction in the compressive strength in the presence of delamination is often associated with progressive local buckling [100]. In WTBs, large and deep delaminations are prone to rapid growth, reducing the compressive strength of the blade due to higher elastic energy that drives the delamination growth [101].
*Shear strength:* The in-plane shear strength is highly influenced by delamination [102]. In addition, delamination reduces the buckling load under in-plane shear. Studies have shown that long, slender [103], and circular delaminations [104] greatly affect the shear strength. A critical failure mode of large WTBs under shear loads is the cross-sectional shear distortion result from the change in the angle of the edgewise load [105]. This mechanism in WTBs can contribute to delamination growth and premature shear buckling.
*Buckling strength:* Delamination greatly affects the buckling strength of composite laminates under compressive loads [71,82]. Delamination results in the formation of sublaminates by separating adjacent layers [76]. Also, delamination size and depth affect the buckling mode. For a WTB, the two critical buckling modes are (I) global–local, induced by a large delamination close to the middle of the laminate [76,106], and (II) local buckling, induced by a large delamination close to the free surface under compressive load [72,107]. The formation of both buckling modes in a WTB has serious consequences on delamination growth (buckling-driven delamination) and the load-carrying capacity of WTBs.
*Fatigue strength:* Delamination growth under fatigue loads is very complex, and many aspects of this phenomenon are not well-captured [85]. The literature reports an 18–23% decrease in the failure stress level of composite laminates in case of minor delamination [92,108]. Furthermore, delamination growth depends on the applied load direction, e.g., under tension fatigue loads, delamination can propagate from the near-surface regions of the composite laminate due to the induced intralaminar damage [109]. Under compression fatigue loads, due to buckling, delamination extends normal to the load direction [110]. The literature further reports a notable reduction in stiffness compared to the strength under fatigue loads in the presence of delamination [92]. This highlights the importance of quantifying the residual stiffness in fatigue loads, as the loss of blade stiffness leads to blade collision with the tower.

### 2.5. Summary of Discussed Key Elements in DTD of Composite Laminates for WTBs

Table 2 summarizes the key elements of DTD of composite laminates in WTBs based on the discussion made in this section. Note that Table 2 only addresses the key elements 1, 2, and 5 discussed in Section 2.1 and illustrated in Figure 5. While the remaining key elements, including stress analysis (key element 3), post-damage loading (key element 4), and design and optimization (key element 6), are critical to DTD, their inclusion requires a more in-depth and extensive analysis of the published literature with revised assumptions and detailed examination of interacting complex mechanisms that are beyond the scope of this review. Table 2 outlines each discussed key element, its driving variable(s), and evaluation methods. As discussed, transverse impact load is the critical load for composite laminates. Note that the drop-weight impact test is the preferred test method for composite laminates exposed to LVI, as it helps replicate the boundary conditions and expected EAMs during a transverse LVI load [73,93]. During the impact load, three major EAMs come into play: matrix cracking, delamination, and fiber breakage. For the purpose of this review, we have focused on the literature that identifies delamination as the major contributor to the damage size and residual characteristics. An interesting observation in the residual characteristics is the significance of shear and flexural residual strengths, which are absent in the current standard [86]. This observation highlights the potential need to further consider those critical residual strengths in the DTD of composite laminates for WTBs.

**Table 2:** Summary of the discussed key elements in DTD of composite laminates for WTBs (adopted from [17]). *R* is the minimum to maximum fatigue stress ratio.

Key Element ^⋄^	Element	Variable	Evaluation
1	Critical load	Transverse impact load	Drop-weight impact test
2	Major energy absorption mechanisms	Matrix cracking Delamination Fiber breakage	NDT, e.g., acoustic emission
	Damage size	Delamination size	NDT, e.g., C-Scan
5	Residual characteristics	Flexural * Compression Shear * Buckling	Coupon testing
		Fatigue	Coupon testing ^†,§^: 1. Tension-compression (*R* = −1) 2. Tension-tension (*R* = 0.1) and compression-compression (*R* = 10)
		Stiffness	1. Deformation analysis ^§^ 2. Full-scale static test ^§^

^⋄^ Key elements 3 (stress analysis), 4 (post-damage loading), and 6 (design and optimization) are excluded due to their complexity and the extensive analysis they require. * Not mentioned in [86]. ^†^ Either method can be used according to [86]. ^§^ See [86] for the requirements and details of evaluation.

## 3. Damage Tolerance of Different Hybrid Lay-Up Configurations: A Qualitative Analysis

This section qualitatively investigates the effect of interlayer hybrid lay-up configurations on the DTD framework outlined in Table 2. The focus is on the major EAMs and damage size induced by LVI, as well as post-impact residual strength. The analysis in this section is limited to interlayer hybrid composites made of two fiber types and does not consider individual fiber behavior. Instead, it treats fibers in a layer as a whole. The strain is assumed uniform in each layer, and there is no stress variation prior to the occurrence of any damage. Furthermore, the fiber content for each type is assumed to be equal within each lay-up configuration, and the total fiber content remains constant across all hybrid lay-up configurations to restrict the analysis to the lay-up design rather than fiber content differences. We further exclude non-hybrid composites to focus on the synergistic effect unique to hybrid composites, which is often overlooked when analyzing hybrid composites.

**Figure 7:**
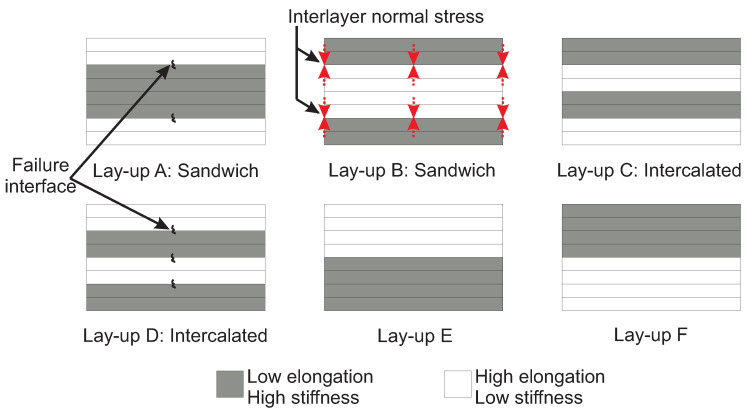
Schematic of different hybrid lay-up configurations of interlayer hybrid composites (rectangles are intended for illustration and do not represent an exact number of layers).

*Different configurations of interlayer hybrid lay-ups:* While various interlayer hybrid lay-ups exist in the literature, the hybrid lay-ups can be broadly classified into six lay-up configurations for the purpose of discussion in this review, as illustrated in Figure 7. HE-LS fibers, e.g., glass are shown in white, while LE-HS fibers, e.g., carbon, are shaded gray. This classification is done so that some potential trends can be qualitatively described, and a generic comparison can be made using different papers surveyed as a part of this review paper.

*Lay-up A* is a sandwich lay-up where HE-LS fibers are placed on the outermost part of the hybrid composite laminate, and LE-HS fibers are located in the interior. *Lay-up B* is also a sandwich lay-up; however, LE-HS fibers are placed on the outermost of the hybrid composite, and HE-LS fibers are in the interior. *Lay-up C* is an intercalated lay-up where LE-HS fibers are placed on the top surface, and HE-LS fibers are located at the bottom. *Lay-up D* is another intercalated lay-up where HE-LS fibers are placed on the top surface, and LE-HS fibers are used at the bottom. No name convention is used for lay-up E and F in the literature; hence, they are only referred to as *Lay-up E* and *Lay-up F* in Figure 7. In lay-up E, several layers of HE-LS fibers are stacked together on the top half of the hybrid laminate, while LE-HS fibers are layered at the bottom half. Lay-up F includes LE-HS fibers on the laminate top half and HE-LS fibers at the bottom half.

### 3.1. Effect of Hybrid Lay-Up Configuration on EAM Under LVI

**Figure 8:**
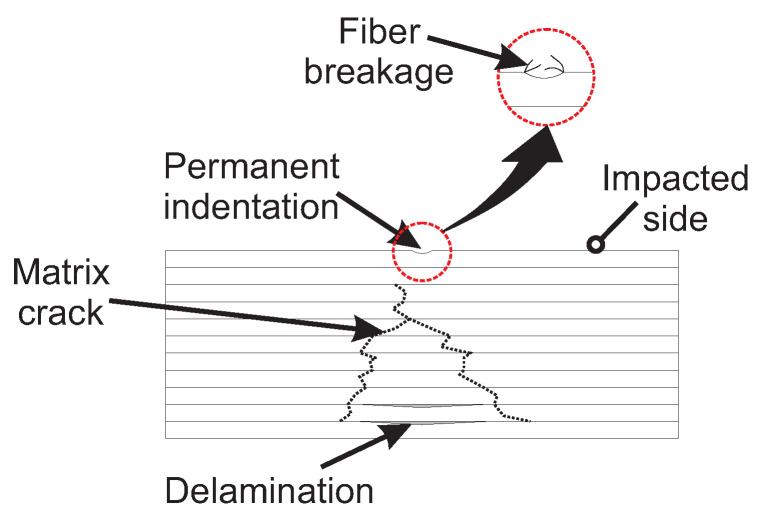
Schematic of the most frequent EAMs in a composite laminate subjected to LVI (adapted from findings in [111,115,116]).

A complete list of reported EAMs of hybrid composites under LVI in the surveyed literature is provided in Table A4 and Table A5 in Appendix B. As can be seen, matrix cracking, delamination, and fiber breakage are the most frequent EAMs under LVI. An schematic of these EAMs is illustrated in Figure 8. Note that a composite with higher absorbed energy is more likely to undergo damage and lose its load-carrying capacity after impact [111]. A suitable hybrid lay-up should allow fibers to reach their ultimate strength without failing prematurely and permit delamination before fiber breakage to prevent catastrophic failure [23,112,113]. Consequently, this would lead to enhanced contact time and increased elastic energy and the impact energy would dissipate through elastic rebounding and rigid body motion rather than damage [77,79,84,114].

Due to the placement of LE-HS fibers on the impacted side, it is expected that the majority of the impact energy is absorbed through fiber breakage in hybrid lay-ups B, C, and F. Furthermore, due to the dominance of fiber breakage, such hybrid lay-ups potentially lead to poor impact behavior and localized permanent indentation on the impacted side [43,117,118,119]. Conversely, hybrid lay-ups A, D, and E with layers of HE-LS fibers on the impacted side may dissipate a large portion of the impact energy through elastic rebounding. This could reduce the possibility of fiber breakage of LE-HS fibers under LVI [40,41]. Among these, lay-up A may be more favorable over lay-ups D and E, as it is expected to minimize the fiber breakage of LE-HS fibers by protecting them between HE-LS fibers [120].

### 3.2. Effect of Hybrid Lay-Up Configuration on Damage Size Under LVI

As discussed in Section 2.4, damage size greatly affects the post-impact residual properties of composites, where a smaller damage size is desirable. Studies show that placing HE-LS fibers on the outermost regions of the hybrid composites can potentially mitigate through-the-thickness damage propagation by confining the damage at the impact zone and reducing delamination at the laminate back face, leading to a reduced damage size [79,121,122]. Therefore, it can be argued that lay-up A may lead to a smaller damage size compared to lay-up B. Comparing lay-ups B and C, the literature [79] suggests that the intercalated configuration of lay-up C may potentially lead to a smaller damage size. A comparative study between lay-up C and D reveals a smaller damage size for lay-up D [123]. Nevertheless, the underlying mechanisms leading to the smaller damage size of lay-up C remain obscure. Given the current scope of research, no definitive conclusion can be made for the damage size of lay-ups E and F. Therefore, further research is necessary to identify the complexities of these configurations and their effect on the induced damage size under LVI.

### 3.3. Effect of Hybrid Lay-Up Configuration on Post-Impact Residual Strength

It is widely accepted that the most affected post-impact residual property is the compressive strength (refer to [71] for a comprehensive discussion). Therefore, most residual strength studies and standards are focused on compression after impact, e.g., [124]. Flexural strength is another residual property measured in the literature, although no standard or procedure is developed for the determination of flexural strength after impact. A comprehensive discussion on the limitations of the present methods for the determination of flexural strength after impact is given in [125]. Due to the lack of literature on the effect of hybrid lay-up on the residual shear, buckling, and fatigue strength of hybrid composites, this section only reviews the effect of hybrid lay-up on the post-impact residual compressive and flexural strength.

**Figure 9:**
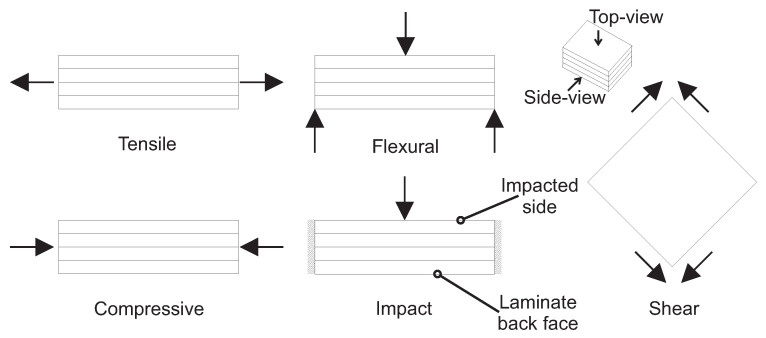
A schematic of a composite laminate subjected to tensile, flexural, compressive, impact (side-view), and shear (top-view) loads (solid arrows indicate the direction of the applied load).

*Flexural residual strength:* A comparative study between lay-ups A and D tentatively suggests a smaller percentage loss in the residual flexural strength for lay-up D. It is postulated that the interface between LE-HS and HE-LS fibers on the tension side of lay-up A (see Figure 9 for directions of applied load) is prone to the formation of long delamination after impact, potentially reducing the residual flexural strength. Although lay-ups C and D might be susceptible to post-impact delamination at the LE-HS and HE-LS fiber interfaces, their intercalated configuration could lead to small delaminations [77,126,127]. The literature suggests a lesser reduction in residual flexural strength for lay-up A compared to lay-up B, possibly due to the presence of HE-LS fibers on the compression side, which may resist delamination-driven buckling [120]. According to LVI failure analysis in Section 4, it can be hypothesized that the induced delamination at the interface of dissimilar fibers together with the failure of LE-HS fibers at the laminate’s back face, may lead to poor residual flexural strength in lay-up E. Also, due to the potentially catastrophic failure, lay-ups E and F are expected to demonstrate negligible residual flexural strength compared to other lay-ups, though conclusive evidence needs to be established for both lay-ups E and F.
*Compressive residual strength:* The literature suggests a more pronounced reduction in residual compressive strength of lay-up B relative to lay-up A, potentially due to the loss of load-carrying capacity from predominant LE-HS fiber breakage during LVI [120,128]. It can be speculated that in the event of impact-induced delamination at the HE-LS and LE-HS interface, the buckling of LE-HS fibers can be mitigated by HE-LS fibers on the outermost of lay-up A. Hence, it could be expected that lay-up A may perform better in terms of residual compressive strength than lay-up B [35,79,84,129,130]. Despite being potentially susceptible to more numbers of delaminations at the interface of dissimilar fibers in lay-ups C and D, their intercalated configuration can potentially enhance the buckling resistance of LE-HS fibers, suggesting reasonable retention of residual compressive strength [79]. Given the risk of catastrophic failure (see LVI failure analysis in Section 4), lay-ups E and F are expected to exhibit the lowest residual compressive strength among all lay-ups, although this conclusive statement requires further investigation.

Table 3 summarizes the discussion on the effect of hybrid lay-up on the damage tolerance of hybrid composite: EAMs, damage size, and residual flexural and compressive strength. Note that the terms ‘high’, ‘medium’ and ‘low’ in Table 3 indicate the degree of favorability. They are only used to provide a descriptive assessment and comparison among different hybrid lay-ups in this review. Note that this categorization serves as a simplified framework for comparison, though it may not capture the distinction among the lay-ups. As can be seen, lay-ups A and D are highly favorable, outperforming the rest. Lay-up C exhibits a medium favorable performance, and lay-ups B, E, and F are expected to underperform from the damage tolerance perspective, making them potentially low favorable lay-ups for WTBs.

## 4. Failure Analysis of Different Hybrid Lay-Up Configurations: A Qualitative Analysis

This section offers a qualitative analysis of failure modes for different hybrid lay-up configurations under different load conditions, i.e., tensile, flexural, compressive, impact, shear (Figure 9), and fatigue loads. Unlike Section 3, this section investigates how different hybrid lay-up configurations behave under various load conditions, focusing on the detailed progression of damage and damage retaliation mechanisms. Such analysis is essential for the static and fatigue proof of composite structures, as it (I) helps designers utilize lower partial safety factors, (II) ensures the failure is progressive, allowing for detection of damage through NDT methods before a catastrophic event, and (III) reduces the failure of the composites during overloading [68].

**Figure 10:**
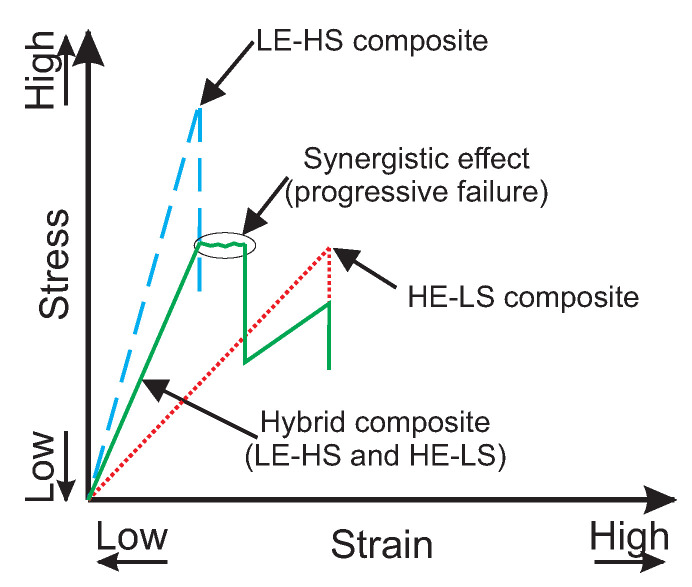
Schematic stress-strain curve representation of LE-HS, HE-LS, and hybrid composites and the expected role of the synergistic effect in delaying the failure of the hybrid composite (adapted from [32]).

Figure 10 illustrates how fiber hybridization in the context of damage tolerance can potentially improve the material’s ability to resist damage and delay failure. This is evident in the schematic of the stress-strain curve, where the LE-HS composite exhibits superior strength and stiffness but lower failure strain. The HE-LS composite shows lower strength and stiffness but a relatively higher failure strain than the LE-HS composite. The hybrid composite combines these attributes, exhibiting an enhanced failure strain compared to the LE-HS composite and increased strength and stiffness compared to the HE-LS composite [131].

Several hypotheses have been proposed to explain the observed increase in the failure strain of hybrid composites [32]: (I) Residual stress induced by different fibers’ coefficient of thermal expansion can modify the strain state within the hybrid composites but are generally considered minimal and inadequate alone to account for the improved failure strain [132]. (II) The interaction between fibers with different elongation capacities and change in the failure development—influenced by statistical distribution—can alter the damage evolution, enhancing the failure strain [133]. (III) The role of dynamic stress concentration, although less explored, suggests that different mass per unit length of fibers leads to out-of-phase stress waves generated by fiber failure, leading to decreased stress concentrations and increased failure strain [134]. These hypotheses, particularly the second, align with the observed synergistic effects, e.g., fiber bridging [135], or buckling-resistance mechanism [136], leading to a progressive failure. The progressive failure of the hybrid composite offers safety benefits and mitigates the risk of catastrophic failure, thereby enhancing the safety and reliability of composite structures in operation [68].

*Tensile failure analysis:* The literature suggests that the failure of LE-HS fiber can potentially bridge the crack faces of HE-LS fibers (fiber bridging) and slow down the induced delamination under tensile load [55,135,137]. Fiber bridging is an intrinsic phenomenon due to crack propagation, i.e., delamination across the reinforcing fibers in composites [138]. Therefore, lay-up A is expected to effectively delay the failure of the hybrid composite. Lay-up B may not be a favorable choice for the progressive failure under the tensile load due to the placement and seemingly negligible role of LE-HS fibers in fiber bridging. The literature suggests an inferior performance in terms of damage-arresting features for lay-ups C and D compared to lay-up A. Within intercalated lay-ups C and D, the failure of LE-HS fibers may induce more failure interfaces between dissimilar fibers (three in lay-ups C and D compared to two in lay-up A), as illustrated in Figure 7, making them susceptible to catastrophic failure [139]. Additionally, in lay-ups C and D, the failure of LE-HS fiber is suspected to induce regions of high-stress concentration, potentially promoting the premature failure of HE-LS fiber [140,141]. It is presumed that lay-ups E and F may demonstrate a progressive failure relative to lay-ups C and D. The apparent benefit of lay-ups E and F lies in placing fibers of the same type on either the top or bottom half of the hybrid laminate. This could potentially (I) mitigate the premature failure of HE-LS fiber due to the high-stress concentration induced by LE-HS fiber failure and (II) reduce the risk of failure associated with the dissimilar fibers at the interface [139]. Note that the advantage of fiber bridging, observed in lay-up A, may not be realized in lay-ups E and F due to the placement of fibers on either the top or bottom half of the lay-up. Therefore, lay-up A could be expected to demonstrate better capability in delaying the failure and is therefore highly favorable.
*Flexural failure analysis:* In flexural load, as illustrated in Figure 9, the composite laminate is subjected to compression (on the top), tension (at the bottom), and shear (between layers) loads [137,142]. It could be argued that the presence of LE-HS fibers on the top region makes lay-up B susceptible to failure under buckling, creating a high-stress region that can potentially propagate through the laminate’s thickness [143]. Therefore, lay-up B is expected to exhibit a catastrophic failure under flexural loads. Given HE-LS fibers on the outermost, which can substantially compress and stretch, lay-up A could potentially demonstrate a progressive failure. This advantage is also shared in lay-ups C and D, where HE-LS fibers are placed on the tension and compression sides, respectively. Nevertheless, distinguishing the failure modes between lay-ups C and D presents challenges [144], although some literature suggests delayed failure for lay-up D [143]. Lay-up E is deemed to show promising delayed failure under flexural load, primarily due to the placement of HE-LS on the compression and LE-HS fiber on the tension side. However, the extent of the progressive failure of lay-up E may be considered moderate. This moderation is due to LE-HS fiber failure on the tension side, which can create a region of stress concentration that may propagate to HE-LS fibers [144,145]. Lay-up F is likely to exhibit a catastrophic failure compared to other lay-ups, mainly because of early buckling failure of LE-HS fibers on the tension side, creating cracks that may easily propagate to HE-LS fibers on the tension side [144,146]. Therefore, lay-up F is deemed unfavorable for the flexural load.
*Compressive failure analysis:* The literature suggests that the interlayer normal stress (red dotted arrows in Figure 7) at the interfaces of LE-HS and HE-LS fibers is prone to delamination and subsequent buckling in the hybrid composite. Therefore, it could be inferred that lay-ups that postpone the buckling of LE-HS fibers may exhibit a progressive failure [35]. This observation is further supported in the literature [137,147,148,149]. Hence, lay-up A is presumed to prevent the buckling of LE-HS fibers by placing them between HE-LS fibers, delaying the failure of the hybrid composite. Lay-ups C and D could potentially show comparable compressive failure but with limited performance compared to lay-up A, making them less favorable than lay-up A. This can be hypothesized as not all LE-HS fibers are supported by HE-LS fibers. The failure of lay-ups B, E, and F is anticipated to be catastrophic or with negligible progressive failure, primarily due to the lack of buckling-resistance support from HE-LS fibers, in which the compressive load suddenly transfers to HE-LS fibers after the buckling of LE-HS fibers.
*LVI failure analysis:* During LVI, the impacted side of the laminate undergoes high compressive stress while the laminate back face experiences tension and large deformation. Placing LE-HS fibers on the impacted side is likely to result in most of the impact energy being absorbed through fiber breakage and induce high localized stress at the impact zone [111,150]. Conversely, placing HE-LS fibers on the impacted side presumably allows stress to be redistributed to areas that can undergo large deformation with minimum fiber breakage [114,128,151]. Therefore, lay-ups B, C, and F, which position LE-HS fibers on the impacted side, might be less favorable for LVI. Lay-up D is suggested to exhibit improved damage-arresting features compared to lay-up A, as its intercalated configuration potentially introduces a crack-arresting mechanism feature, delaying the transverse shear crack propagation across dissimilar layers under LVI [130]. However, some studies report enhanced impact resistance for lay-up A as (I) the failure of LE-HS fibers can be potentially mitigated by placing them between HE-LS fibers and (II) the risk of failure for LE-HS fibers on the laminate back face, subjected to high tensile stress, can be largely minimized (see Figure 9) [120]. Therefore, we assume the effectiveness of lay-ups A and D in postponing failure under LVI comparative. Lay-up E may be less favorable than lay-up D, as lay-up E may not demonstrate a progressive failure. It can be assumed that in case of the failure of LE-HS fibers in lay-up E, the stress could suddenly transfer to HE-LS fibers on the top half, leading to the catastrophic failure of the hybrid composite [152].
*Shear failure analysis:* Literature on the shear assessment of interlayer hybrid composites is limited [153,154], and studies on the interlayer hybrid lay-up assessment under in-plane shear load are scarce [155]. Therefore, a rigorous comparison of the shear failure modes of the hybrid lay-ups based on the available literature is challenging. Furthermore, the current standards are inapplicable to hybrid composites under shear load, further complicating the obtained failure modes and subsequent failure analysis of hybrid composites [156]. A comprehensive discussion can be found in [102]. Therefore, more research is needed to understand the effect of hybrid lay-up on the in-plane shear of interlayer hybrid composites.
*Fatigue failure analysis:* Despite the research on the fatigue of interlayer hybrid composites, e.g., [49,157,158], the comparison among hybrid lay-ups is limited to fatigue behavior, e.g., S-N (stress-number of cycles) diagram [159]. Typically, the literature does not compare the failure modes among hybrid lay-ups. Furthermore, the dependency of the fatigue behavior and failure modes on the load direction and hybrid lay-up configuration makes the comparison more complicated. Nevertheless, the literature suggests that the failure of LE-HS fibers can affect the stress distribution in surrounding fibers, facilitating the premature failure of HE-LS fibers under tension-tension cyclic load [160]. Therefore, it can hypothesized that a hybrid lay-up that delays the failure of LE-HS fibers and subsequent failure of HE-LS fibers may lead to a progressive failure under fatigue. However, assessing this statement requires further investigation under different load directions.

Table 4 summarizes the discussion on the failure analysis under different load conditions for the proposed hybrid lay-up configurations in Figure 7. The result indicates lay-up A outperforms the rest of the lay-ups in utilizing the synergistic effect that could potentially delay the failure of interlayer hybrid composites; therefore, it is highly favorable for WTBs. Lay-up D shows competitive results to lay-up A, making it a medium favorable choice for WTBs. Lay-ups C and E exhibit a potential capacity for progressive failure through the synergistic effect, though they are less favorable for LVI (lay-up C) and compressive loads (lay-up E). Overall, lay-ups B and F are low favorable hybrid lay-ups, as they do not benefit from the synergistic effect to delay the failure of the hybrid composite.

## 5. Mechanical Properties of Hybrid Composites: A Quantitative Analysis

This section aims to quantitatively investigate the effect of interlayer fiber hybridization on the mechanical properties with an explicit focus on the tensile and flexural properties for WTBs. Given the operational and structural requirements, the materials for WTBs require high strength and stiffness to maintain structural integrity and aerodynamic performance [161]. While the impact toughness is acknowledged as a critical factor in the literature, its sole use as an indicator for hybrid effect cannot be used in the context of this review due to (I) dependency of impact toughness and induced EAMs on the initial impact energy [56,120,127,162,163] and (II) complexity in interpreting the hybrid effect under impact load. For example, a low-impact toughness could be due to the synergistic effect (localized elongation and elastic rebounding) [112] or voids and non-wetted areas at the interface [30,164,165]. Furthermore, this section does not examine individual hybrid composite behavior but reviews the primary mechanisms leading to hybrid effects.

### 5.1. Methodology

*Assumptions:* The following assumptions are made to facilitate combining and comparing various data in the literature.

1. Various types in a group of fiber are categorized under a name that represents that group of fiber. For example, various glass (E, S, L) and Kevlar (Kevlar-29, Kevlar-49) fibers are grouped as glass and aramid fibers, respectively.
2. FVF is calculated assuming zero void.
3. The effect of stacking sequence, fabric construction, resin type, and sizing are not considered in the analysis.
4. Similar fiber distribution and resin impregnation are assumed for all composites, regardless of their manufacturing method.
5. The uncertainties due to fiber and resin manufacturing, production batch, storage, test method, testing machine, lab environment, operator skill, and measurement error are not considered.
6. The data include tensile test specimen sizes ranging from 12 × 100 mm^2^ to 29 × 246 mm^2^ and flexural test specimen sizes varying from 8 × 95.5 mm^2^ to 60 × 180 mm^2^. To facilitate a comparative analysis from a wide range of hybrid composites, variations in test coupon size, although present, are not considered. Error bars, elaborated in the subsequent section, are used to account for the data variability and enhance the interpretation of the result.

*Method:* This review includes the synthetic fibers commonly used in the literature: glass, carbon, and aramid. The selection of natural fibers covers a diverse range of existing literature to identify potential candidates for hybridization and alternatives to glass composite. Existing literature with reported tensile and/or flexural properties for both hybrid and non-hybrid composites are examined to extract the following mechanical properties: (I) tensile strength, (II) tensile modulus, (III) flexural strength, and (IV) flexural modulus. The collected data are labeled into five categories: natural (N), synthetic (S), natural/natural (NN), synthetic/natural (SN), and synthetic/synthetic (SS), where only the last three are termed as hybrid composite (Figure 3). The mechanical properties in each label are then averaged to facilitate the comparison between hybrid and non-hybrid composites. In addition, the following method is utilized to identify alternative hybrid composites to the glass composite: (I) The average tensile and flexural properties for each hybrid composite are calculated. (II) A score of one is given to each property (e.g., tensile strength) of a hybrid composite that is found to meet or exceed its corresponding property of the glass composite. Otherwise, a score of zero is given. (III) The scores for each property are summed, ranging from zero (none of the properties met) to four (all the properties met or exceeded). (IV) Hybrid composites with a total score of four are recognized as potential alternatives to the glass composite.

*Error bar:* The standard error of the mean (SEM) is only used for representing error bars in this review and is defined according to Equation (1).

(1) $$\mathrm{SEM}=\frac{\mathrm{SD}}{\sqrt{n}}$$

where SD is the standard deviation, and *n* is the number of samples. SEM is useful when the variation in data is large. Using SEM, two criteria need to be reported: (I) clearing stating that the error bars represent SEM and (II) the number of samples in each category [166]. SD can be calculated using Equation (1) in all the plots in this review. The number on the top of each bar chart indicates the average. In this review, the number of samples and SEM values per label are provided in a table next to the plot. An overview of the data, including the literature and a list of fibers, resins, and hybrid lay-ups, is given in Appendix B.

### 5.2. Mechanical Properties of Hybrid and Non-Hybrid Composites

**Figure 11:**
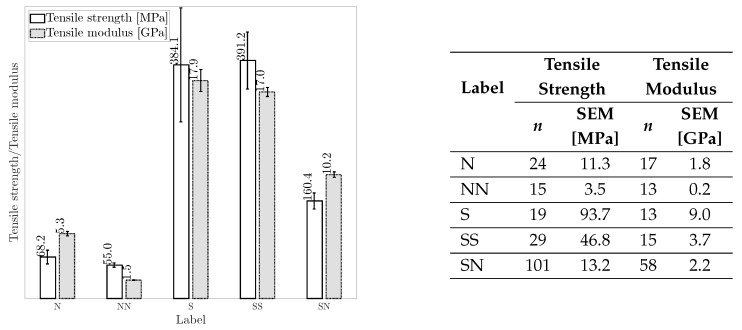
Tensile properties of hybrid and non-hybrid composites (the average value is given at the top of each bar chart and the error bars indicate the standard error of the mean (SEM). The number of samples, *n* and SEM for each label is given in the table).

**Figure 12:**
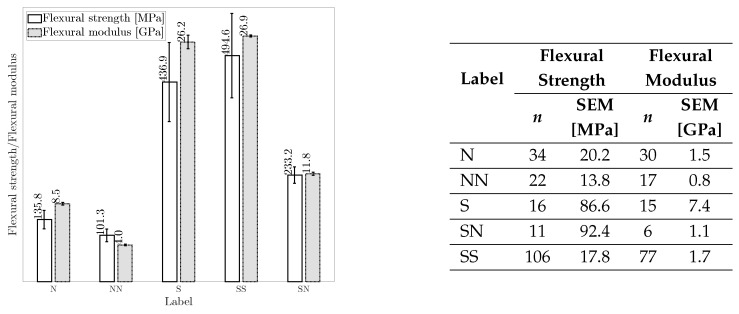
Flexural properties of hybrid and non-hybrid composites (the average value is given at the top of each bar chart and the error bars indicate the standard error of the mean (SEM). The number of samples, *n* and SEM for each label is given in the table).

*Tensile properties:* Figure 11 shows the tensile properties of hybrid and non-hybrid composites. Note that the results in Figure 11 (and later in Figure 12) are presented in their reported units (MPa and GPa) without normalization by any parameters, e.g., density to highlight the intrinsic effect of fiber hybridization on the mechanical properties. As can be seen, SS and S composites exhibit the highest average tensile properties, with SS composites offering slightly higher tensile strength and lower tensile modulus than S composites. The slight improvement in the tensile strength of SS composite can be attributed to: (I) the synergistic effect (i.e., carbon fibers provide high strength while glass fibers facilitate high strain capacity) and (II) enhanced stress distribution, which can absorb and redistribute stress under tension more effectively [33,167]. In comparison to S, SN composites demonstrate lower tensile properties. Due to their inherent entanglement and different diameters, natural fibers are more difficult to orient, reducing the mechanical properties of SN composites [165,168]. Furthermore, non-uniform stress transfer to unfailed fibers leads to the inferior tensile properties of SN composites. This phenomenon arises due to the different tensile strain-to-failure and modulus between the synthetic and natural fibers. High-stress regions can be formed in case of early failure of synthetic or natural fibers, redistributing the stress among unfailed fibers and facilitating the failure of SN composites [139,169,170].

N and NN composites offer inferior average tensile properties among all composites, with NN composites exhibiting the lowest tensile properties. The higher tensile properties of N composites can be attributed to fewer compatibility issues between fiber and resin [171]. Furthermore, surface flaws, non-wetted areas [172,173], and moisture absorption of natural fibers are aggravated in NN composites due to the inclusion of different natural fibers, leading to lower tensile properties compared to N composites [174,175,176]. Conversely, SN composites demonstrate enhanced tensile properties compared to N composites, primarily due to the presence of synthetic fibers with higher tensile strength and modulus and their enhanced interfacial bonding with polymeric resins [80,150,175,177].

*Flexural properties:* Figure 12 shows the flexural properties of hybrid and non-hybrid composites. SS composites exhibit the highest average flexural properties, followed by S composites. Two major mechanisms contribute the most to the improved flexural properties of SS composites: (I) the inclusion of two high-strength and modulus synthetic fibers and (II) the placement of high-strength fibers at a distance from the neutral axis [142]. The lower flexural properties of SN composites compared to S composites are because of the inclusion of natural fibers with inferior mechanical properties and poor fiber orientation. Also, due to different tensile and compressive properties, the early failure of natural or synthetic fibers leads to out-of-plane normal cracks that grow at the interface of both fibers, creating high-stress regions that contribute to the rapid failure and lower flexural properties of SN composite than S composite [112,139].

N and NN composites have the lowest flexural properties, mainly due to the weak interfacial bonding of natural fibers [178] and their poor compressive and tensile properties [179]. The lower flexural properties of NN composites compared to N composites can be attributed to the different failure modes of natural fibers within NN composites under flexural load. Typically, the outermost regions of the non-hybrid composites are the first areas that undergo failure due to the high stress under flexural load. However, the failure in NN hybrid composites can be initiated because of delamination at the interface of dissimilar fibers, leading to their lower flexural properties [142,171]. The improvement in the flexural properties of SN composites in comparison to N composites is due to synthetic fibers with higher compressive properties [180,181] and better fiber orientation [80,165], which leads to enhanced flexural properties of SN composites.

*FVF and fiber hybridization:* Table 5 presents the average FVF and percentage changes in the tensile and flexural properties of hybrid composites (NN, SN, SS) relative to non-hybrid composites (N, S) as the baseline. For each composite label, FVF is first averaged separately for the literature reported FVF and one of the corresponding mechanical properties, including tensile strength, tensile modulus, flexural strength, and flexural modulus. Then, these four averaged FVFs, each associated with one of the mechanical properties, are further averaged to represent averaged FVF. Despite similar FVF, NN composites show decreased tensile and flexural strength with a notable drop in the tensile and flexural modulus compared to N composites. This suggests that, on average, the hybridization of natural fibers (NN composite) may lead to a hybrid composite with inferior tensile and flexural properties than N composite. In contrast, SN composites, with a 35% FVF (consisting of 19% natural and 16% synthetic fibers on average), exhibit considerable improvements in the tensile and flexural strengths and moduli compared to N composites. This indicates that introducing synthetic fibers to replace a portion (approximately 16% on average) of natural fibers in SN composites can substantially improve their tensile and flexural properties compared to N composites. This finding highlights the potential benefits of carefully balanced fiber contents in enhancing the tensile and flexural properties of SN composites.

The comparison between SN and S composites in Table 5 reveals a remarkable drop in the tensile and flexural properties of SN composites. This suggests that a higher portion of natural fiber (19%) relative to synthetic fibers (16%), on average in SN composites, can adversely affect their tensile and flexural properties. This observation aligns with the literature, emphasizing the importance of determining an optimum natural fiber content in SN composite [106]. This optimum corresponds to natural fiber content at which the tensile and flexural properties maximize before a decline is observed. This optimum is determined by testing SN composites with different natural fiber content [106]. Interestingly, Table 5 shows minor differences in the tensile and flexural strengths and moduli of S and SS composites despite SS composites having higher FVF. Therefore, it could be inferred that for the same FVF, SS composites, on average, may demonstrate lower tensile and flexural properties than S composites. While this observation may appear generally applicable, further research is needed to assess its validity.

*Hybrid effect:* Table 6 summarizes the hybrid effect by comparing the average tensile and flexural properties of hybrid and non-hybrid composites in Figure 11 and Figure 12. Within the context of this review, if a hybrid composite’s mechanical property deviates by more than 10% from its non-hybrid composite as the baseline, it is labeled as a positive hybrid effect (+) for exceeding and a negative hybrid effect (−) for being less. ∼ is used for minor differences within 10% to show negligible differences between the mechanical properties of the hybrid composite and its non-hybrid baseline. Table 6 shows that the determination of a positive or a negative hybrid effect depends on the baseline for comparison. For example, for the tensile and flexural properties, SN composites show positive and negative hybrid effects in comparison to N and S composites, respectively. Also, NN composites demonstrate a negative hybrid effect in the tensile and flexural properties compared to N composites. SS composites, except for the flexural strength, exhibit negligible improvement in their tensile and flexural properties in comparison to S composites. In summary, a conclusion on the hybrid effect depends not only on the fibers involved in the hybridization but also a clear statement of the baseline is essential.

### 5.3. Alternative Hybrid Composites to Non-Hybrid Glass Composite

According to Section 5.2, SS composites are the only potential hybrid composites that provide competitive mechanical properties compared to S composites. Nevertheless, in addition to the list of SS composites, a comprehensive list of SN composites is provided to explore their individual potential as alternative composites to the glass composite.

**Figure 13:**
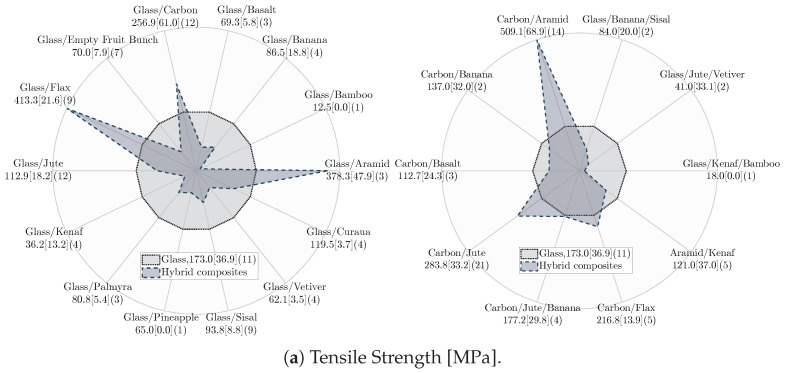

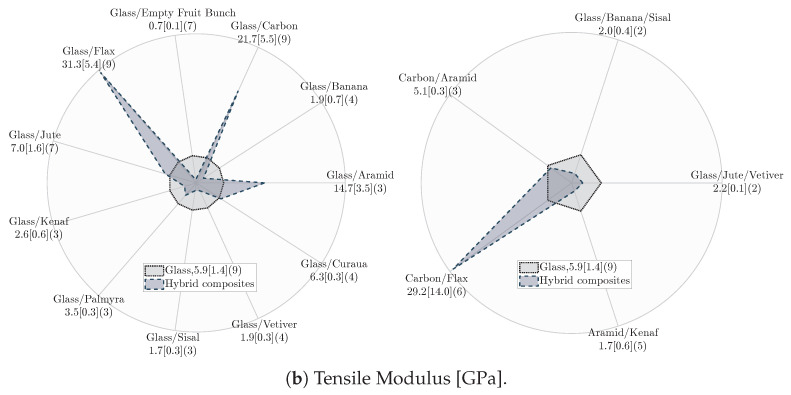
A comparison between (**a**) tensile strength and (**b**) tensile modulus of glass and other hybrid composites. The first number indicates the average tensile property, followed by the standard error of the mean (SEM) in brackets and number of samples in parenthesis (MATLAB code obtained from [190]).

**Figure 14:**
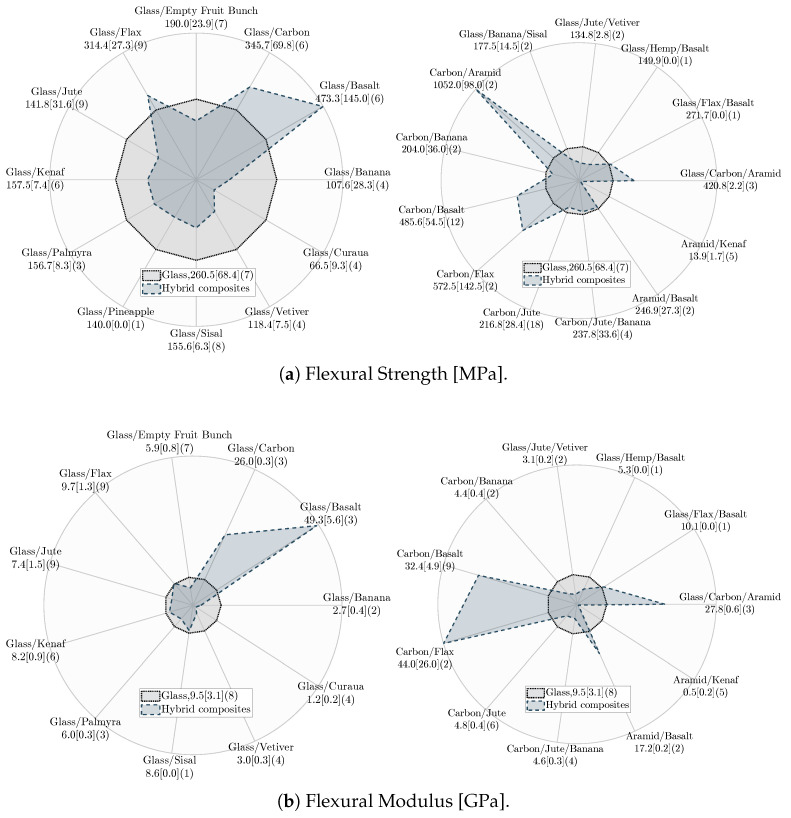
A comparison between (**a**) flexural strength and (**b**) flexural modulus of glass and other hybrid composites. The first number indicates the average flexural property, followed by the standard error of the mean (SEM) in brackets and number of samples in parenthesis (MATLAB code obtained from [190]).

Figure 13a,b compare the average tensile strength and modulus of several hybrid composites and the glass composite. In general, it can be seen that SS hybrid composites (i.e., glass/carbon, glass/aramid, carbon/aramid) and a few SN composites (i.e., glass/flax, carbon/flax, carbon/jute, and carbon/jute/banana) provide superior tensile properties compared to the glass composite. A comparison of the flexural strength and modulus among hybrid composites and the glass composite is provided in Figure 14a,b. Overall, the abovementioned SS hybrid composites and hybrid glass/flax, carbon/flax, glass/basalt, carbon/basalt, glass/basalt, and glass/flax/basalt composites lead to superior flexural properties than the glass composite. A common observation in the abovementioned hybrid composites is the presence of SS composites, glass/flax, and carbon/flax with improved tensile and flexural properties than the glass composite. The relatively low mechanical properties of the glass composite reported in Figure 13 and Figure 14 are due to (I) the use of woven glass textile in reviewed literature (such as in [151,177,182,183,184,185,186]), (II) a range of varying FVF in the order of 9–60% reported in the literature (such as in [184,185,186,187,188]) compared to the 50–60% FVF in standard WTBs [189], and (III) lack of fiber alignment in the loading direction considered in [165,182,187,188]. However, it is essential to note that literature uses the same glass fiber to compare performance for both hybrid and non-hybrid glass composite. This uniformity still ensures the validity of our comparative study and provides insight into identifying the potential alternatives to the glass composite. A more comprehensive discussion on potential hybrid composites that are alternative to glass composites is provided below.

Table 7 shows the score table for the tensile and flexural strength and modulus of hybrid composites in Figure 13 and Figure 14. Hybrid glass/carbon composite with a total score of 4 is a high potential alternative to glass composite, owing to the combination of high strength and modulus carbon fibers and long elongation of glass fibers [33,116,167]. Hybrid carbon/flax and glass/flax obtain a total score of 4, making them potential hybrid composites to replace the glass composite. The good mechanical properties and interfacial bonding of flax fibers and the superior mechanical properties of the glass and carbon fibers lead to improved tensile and flexural properties of their hybrid composites [150,175,177,191]. Flax fibers are introduced as alternatives to glass fibers in the literature, mainly due to the higher specific tensile and flexural properties of flax fibers [21,192,193]. Despite their potential, the application of flax fibers in WTBs introduces challenges: their hydrophilic nature (which can negatively affect their mechanical properties) [194,195], variability in mechanical properties (due to growth conditions and processing methods) [196], and the logistics of scaling up flax fiber production to meet industrial demand. Additionally, the different operational environments of WTBs—ranging from onshore to offshore and tropical to cold climate—could accelerate the moisture absorption and degradation of mechanical properties of flax fibers. Therefore, further research is needed to assess the feasibility of using flax fibers in WTBs. The low compressive strength, low adhesion to polymeric resins, moisture absorption, and degradation upon exposure to ultraviolet radiation of aramid fibers make hybrid carbon/aramid, glass/aramid, and glass/carbon/aramid composites inappropriate choices for WTBs [16]. In addition to offering a lower tensile strength than glass composite, the high density of basalt fibers makes hybrid carbon/basalt, glass/basalt, and glass/flax/basalt composites unfavorable choices for WTBs [197,198]. Other hybrid composites lead to inferior tensile and flexural properties in comparison to the glass composite, making them unfavorable for WTBs.

## 6. Conclusions

This paper is the first attempt to systematically analyze the application of interlayer hybrid fiber composites for wind turbine blades (WTB). Interlayer hybrid fiber composites are easy to manufacture and combine the advantage of low elongation (LE)-high stiffness (HS) fibers, e.g., carbon and high elongation (HE)-low stiffness (LS) fibers, e.g., glass. Such a strategic combination offers enhanced impact resistance, residual strength, and reduced delamination size compared to non-hybrid composites. This review outlines a damage tolerance design (DTD) framework of composite laminates for WTBs with an emphasis on the transverse impact load and categorizes interlayer hybrid lay-up into six different configurations for a structured discussion. Our qualitative and quantitative analyses assess the damage tolerance and failure analysis across these configurations and compare tensile and flexural properties of hybrid and non-hybrid composites. Conclusions, based on the surveyed literature, are summarized as follows:

1. The discussed DTD framework reveals that in addition to the compression and buckling residual strengths mentioned in the standard (DNVGL-ST-0376) [86], the flexural and shear residual strengths of composites are required to be evaluated as the residual characteristics.
2. A qualitative analysis among different interlayer hybrid lay-ups shows that a sandwich lay-up with HE-LS fibers, e.g., glass on the outermost and LE-HS fibers, e.g., carbon on the innermost regions of an interlayer hybrid composite leads to the best compromise between the impact behavior and underlying failure modes leading to a progressive failure for WTBs.
3. The quantitative analysis indicates that the tensile and flexural properties of natural fibers can be effectively enhanced upon hybridization with synthetic fibers. Synthetic/natural (SN) composites exhibit the largest improvement in the tensile and flexural properties when compared to natural (N) composites. Furthermore, the result shows that obtaining a positive hybrid effect in synthetic/synthetic (SS) composites (compared to synthetic (S) composites) is directly connected to increasing fiber volume fraction of SS composites.
4. The quantitative analysis also shows that synthetic/natural (SN) and natural/natural (NN) hybrid composites exhibit a negative hybrid effect in the tensile and flexural properties in comparison to non-hybrid S and N composites, respectively. Conversely, a positive hybrid effect for the same properties is observed in SN composites compared to N composites. SS composites show negligible improvement in the tensile and flexural properties compared to S composites. A positive hybrid effect is only observed in flexural strength for SS composites.
5. The quantitative analysis between glass and hybrid composites (SN, SS), based on the tensile and flexural properties in the literature, reveals that hybrid glass/carbon, glass/flax, and carbon/flax composite could be potential alternatives to the glass composite for WTBs.

## 7. Recommendations for Future Work

Below are the recommendations for future work:

1. Stress analysis in the vicinity of the damage is not covered in this review. A better understanding of the stress distribution in the vicinity of damage(s), the level of criticality, and behavior in different hybrid lay-ups is necessary. Future studies should implement different numerical methods, e.g., finite element methods for the stress analysis and predict the mechanical properties and failure of hybrid composites more cost-effectively, especially when the in-situ testing becomes expensive.
2. The literature review shows limited studies on the effect of hybrid lay-up on the in-plane shear, buckling, and fatigue properties and their post-impact residual properties. Future research needs to explore the in-plane shear and buckling assessment of hybrid composites and study the fatigue behavior of various hybrid lay-ups in different load directions. Furthermore, available standards and methods are insufficient to characterize the shear properties of hybrid composites. Future research could involve developing methods to reliably determine the shear properties of hybrid composites.
3. Future research could involve a more comprehensive testing campaign on the mechanical properties and damage tolerance to evaluate the application of hybrid glass/carbon, glass/flax, and carbon/flax for WTBs. Research in hybrid glass/flax and carbon/flax composites should further explore the viability of using flax in WTBs under diverse conditions and climates.
4. Future research requires bridging coupon-scale experiments and full-scale applications, ensuring the observed synergistic effects are scalable and applicable to WTBs.
5. Impregnation ensures resin distribution and fiber wetting, which are critical for achieving the desired mechanical properties of composites. Future studies could address the compatibility of resin systems, the challenges in wetting different fiber types, and effective impregnation strategies to maximize the mechanical properties of hybrid composites.

## Figures and Tables

**Table 1:** Mechanical properties of several common fibers [34].

Fiber	Stiffness * [GPa]	Elongation [%]
Aramid	60	2.5–3.7
Carbon	240–425	1.4–1.8
E-glass	73	2.5
Flax	27.6	2.7–3.2
Sisal	9.4–22	3–7

* Tensile modulus in the fiber direction.

**Table 3:** Summary of the discussion on key elements of damage tolerance of hybrid composite for different hybrid lay-ups. N/A: No available result.

Lay-Up	Energy Absorption Mechanism	Damage Size	Residual Flexural Strength	Residual Compressive Strength	Overall Favorability for WTBs
A	*High*	Smaller than lay-up B	*Medium* (susceptible to long delamination on tension side)	*Medium* (better buckling resistance than lay-up B due to outer HE-LS fiber support)	*High*
B	*Low*	Larger than lay-up A	*Low* (prone to delamination-driven buckling on compression side)	*Low* (lack of buckling resistance mechanism)	*Low*
C	*Low*	Smaller than lay-up B	*High* (limited delamination due to intercalated lay-up)	*Medium* (enhanced buckling resistance)	*Medium*
D	*Medium*	Smaller than lay-up C	*High* (Similar concerns as lay-up C)	*Medium* (Similar concerns as lay-up C)	*High*
E	*Medium*	N/A	*Low* (susceptible to delamination at the interface of dissimilar fibers and fiber failure at laminate’s back face)	*Low* (risk of catastrophic failure ^†^)	*Low*
F	*Low*	N/A	*Low* (risk of catastrophic failure *)	*Low* (risk of catastrophic failure ^†^)	*Low*

* See flexural failure analyses in Section 4. ^†^ See compressive failure analyses in Section 4.

**Table 4:** Summary of the discussion on failure analysis of hybrid composite for different hybrid lay-ups.

Lay-Up	Tension Load	Flexural Load	Compressive Load	LVI	Overall Favorability for WTBs
A	*High* (delayed failure by fiber bridging)	*High* (placement of HE-LS fibers on the outermost)	*High* (buckling-resistance support for LE-HS fibers)	*High* (placement of HE-LS fiber on laminate’s back face, mitigate LE-HS fiber breakage)	*High* (especially if progressive failure and impact resistance are crucial)
B	*Low* (prone to catastrophic failure due to lack of damage-arresting features)	*Low* (susceptible to buckling on the compression side)	*Low* (prone to catastrophic failure due to lack of buckling-resistance support for LE-HS fibers)	*Low* (susceptible to fiber breakage on impacted side)	*Low* (risk of catastrophic failure)
C	*Medium* (prone to catastrophic failure due to more failure interface than lay-up A and high- stress regions)	*Medium* (placement of HE-LS fiber on tension side)	*Medium* (partially buckling- resistance support for LE-HS fibers)	*Low* (similar concerns as lay-up B)	*Low* (requires additional design considerations under LVI)
D	*Medium* (similar concerns as lay-up C)	*Medium* (placement of HE-LS fiber on compression side)	*Medium* (similar concerns as lay-up C)	*High* (enhanced damage- arresting features due to intercalated lay-up)	*Medium* (Limited performance under tensile, flexural, and compressive loads)
E	*Medium* (less prone to premature failure at the interface and induced high-stress regions)	*Medium* (risk of crack propagation due to LE-HS fiber failure on tension side)	*Low* (similar concerns as lay-up B)	*Medium* (sudden stress transfer to HE-LS fibers due to LE-HS fiber failure on tension side)	*Low* (requires buckling- resistance considerations for compressive load)
F	*Medium* (similar concerns as lay-up E)	*Low* (risk of catastrophic failure, early buckling of LE-HS fiber)	*Low* (similar concerns as lay-up B)	*Low* (similar concerns as lay-up B)	*Low* (similar concerns as lay-up B)

**Table 5:** Average FVF of hybrid composites (NN, SN, SS) and the percentage changes in the tensile and flexural properties compared to non-hybrid composites (N, S) as baseline.

Composite	Baseline for Comparison	Average FVF [%]	Tensile Strength (Difference to Baseline [%])	Tensile Modulus (Difference to Baseline [%])	Flexural Strength (Difference to Baseline [%])	Flexural Modulus (Difference to Baseline [%])
N	-	33	-	-	-	-
NN	N	33	−19	−72	−25	−53
SN	N	35 *	+135	+91	+72	+38
S	-	39	-	-	-	-
SN	S	35 *	−58	−43	−47	−55
SS	S	58	+2	−5	+13	+3

* FVF of SN comprises 19% of natural and 16% of synthetic fibers on average.

**Table 6:** A comparison of positive (+) and negative (−) hybrid effects based on the average tensile and flexural properties of hybrid composite (NN, SN, SS) and non-hybrid composites (N, S) in Figure 11 and Figure 12. ∼ shows negligible difference.

Hybrid Composite	Baseline for Comparison	Tensile Strength	Tensile Modulus	Flexural Strength	Flexural Modulus
NN	N	−	−	−	−
SN	N	+	+	+	+
SN	S	−	−	−	−
SS	S	∼	∼	+	∼

**Table 7:** Score table of the tensile and flexural properties of hybrid composites. A score of 0 indicates a mechanical property less than that of the glass composite, while a score of 1 shows that it is equal to or better than the glass composite. N/A: no available data (scored as zero).

Label	Hybrid Composite	Tensile Strength	Tensile Modulus	Flexural Strength	Flexural Modulus	Total Score
SS	Glass/Carbon	1	1	1	1	4
SN	Carbon/Flax	1	1	1	1	4
SN	Glass/Flax	1	1	1	1	4
SS	Carbon/Aramid	1	0	1	N/A	2
SN	Carbon/Basalt	0	N/A	1	1	2
SS	Glass/Aramid	1	1	N/A	N/A	2
SN	Glass/Basalt	0	N/A	1	1	2
SS	Glass/Carbon/Aramid	N/A	N/A	1	1	2
SN	Glass/Flax/Basalt	N/A	N/A	1	1	2
SN	Aramid/Basalt	N/A	N/A	0	1	1
SN	Glass/Curaua	0	1	0	0	1
SN	Carbon/Jute	1	N/A	0	0	1
SN	Carbon/Jute/Banana	1	N/A	0	0	1
SN	Glass/Jute	0	1	0	0	1
SN	Aramid/Kenaf	0	0	0	0	0
SN	Carbon/Banana	0	N/A	0	0	0
SN	Glass/Bamboo	0	N/A	N/A	N/A	0
SN	Glass/Banana	0	0	0	0	0
SN	Glass/Empty Fruit Bunch	0	0	0	0	0
SN	Glass/Kenaf	0	0	0	0	0
SN	Glass/Palmyra	0	0	0	0	0
SN	Glass/Pineapple	0	N/A	0	N/A	0
SN	Glass/Sisal	0	0	0	0	0
SN	Glass/Vetiver	0	0	0	0	0
SN	Glass/Kenaf/Bamboo	0	N/A	N/A	N/A	0
SN	Glass/Jute/Vetiver	0	0	0	0	0
SN	Glass/Hemp/Basalt	N/A	N/A	0	0	0
SN	Glass/Banana/Sisal	0	0	0	N/A	0

## Data Availability

Data will be made available on request.

## Abbreviations

BVID	Barely visible impact damage
DTD	Damage tolerance design
EAM	Energy absorption mechanism
FVF	Fiber volume fraction
HE	High elongation
HS	High stiffness
LE	Low elongation
LS	Low stiffness
LVI	Low-velocity impact
N	Natural
NDT	Non-destructive test
NN	Natural/Natural
OWT	Offshore wind turbine
S	Synthetic
SD	Standard deviation
SEM	Standard error of the mean
SN	Synthetic/Natural
SS	Synthetic/Synthetic
WTB	Wind turbine blade

## Appendix A. Definitions

**Catastrophic failure:** The state of sudden and complete failure of the structure without prior warning or indication.
**Contact time:** Time duration after which there is no contact between the target and the impactor [84].
**Critical size:** The maximum residual strength at limit service load.
**Damage size:** The portion of the laminate that undergoes different energy absorption mechanisms upon impact load. This review defines the damage size as the area formed by delamination.
**Energy absorption mechanism:** Various failure modes or energy dissipation mechanisms in composites during impact load.
**Impact behavior:** Includes different aspects of impact response (contact force, time, and displacement), impact resistance (to damage), and impact damage tolerance (post-impact residual properties) [125].
**Impact toughness:** The ability of the material to absorb the impact energy during an impact event through deformation, fracture, without plastic deformations [199]. In the context of composites, the impact toughness can be defined as the ability of the composite laminate to absorb the impact energy through various energy absorption mechanisms (e.g., matrix cracking and delamination) under impact load.
**Limit service load:** Loads that a wind turbine blade experiences during its lifetime. This load is referred to as characteristic load in [86].
**Progressive failure:** The state where the damage develops slowly over a period of time; hence, the damage can be detected during the detection period.
**Reserve margin:** The residual strength because of the difference between design load and design strength [11].
**Residual strength:** The remaining static strength of the composite laminate or structure at any time during service in the presence of damage [17].
**Structural damage:** Types of damage that compromise the blade’s structural integrity and affect its lifetime by reducing its strength and stiffness [9].
**Sublaminate:** A portion within a composite laminate bounded by a free surface on one side and a delamination on the other side.
**Ultimate load:** Limit service load multiplied by a partial safety factor. This load is referred to as design load in [86].
**Ultimate strength:** Load-carrying capacity of the blade in the absence of damage.

## Appendix B. Data Overview

**Table A1:** List of the literature in the quantitative analysis (*n* denotes the number of data).

Reference	*n*					Reference	*n*				
	**N**	**NN**	**S**	**SN**	**SS**		**N**	**NN**	**S**	**SN**	**SS**
[44]	3					[200]	2	1		6	
[118]	1		1	1		[181]				12	
[182]	1		1	1		[165]			1	6	
[201]	3		1	2		[202]	1			2	
[127]	1		1	2		[188]	1		1	3	
[187]			1	1		[184]	1		1	2	
[203]	2	1				[185]	1		1	2	
[204]		2				[186,205]	1		1	2	
[206]	2	1				[128,177]	2		1	4	
[207]	2	1				[208]	1		1	5	
[209]	1	2				[139]	1			5	
[180]	2	3				[210]	1		1	1	
[178]		6				[211]	7			7	
[212]		1				[213]	1			6	
[214]				3		[215]			2		2
[165]			1	1		[163]					3
[80]	1			3		[146]					3
[20]				6		[183]			3		6
[216]				1		[217]					6
[218]		3		6		[219]			2		10
[220]	1		1	4		[77]	1		1	2	
[170]	1		2	6		[151]	1		2	6	
[142]	4		8			[221]				6	3
[222]				6		[223]	1		1	4	

**Table A2:** List of fibers included in the quantitative analysis (hybrid and non-hybrid composites).

N	NN	S	SN	SS
Bamboo	Jute/Basalt	Aramid	Aramid/Basalt	Carbon/Aramid
Banana	Jute/Empty fruit bunch	Carbon	Aramid/Kenaf	Glass/Aramid
Basalt	Jute/Oil palm	Glass	Carbon/Banana	Glass/Carbon
Curaua	Jute/Palmyra		Carbon/Basalt	Glass/Carbon/Aramid
Empty fruit bunch	Jute/Vetiver		Carbon/Flax	
Flax	Sisal/Bamboo		Carbon/Jute	
Hemp	Sisal/Banana		Carbon/Jute/Banana	
Jute	Sisal/Cotton		Glass/Bamboo	
Kenaf	Flax/Hemp/Basalt		Glass/Banana	
Oil Palm			Glass/Banana/Sisal	
Palmyra			Glass/Basalt	
Sisal			Glass/Curaua	
			Glass/Empty fruit bunch	
			Glass/Flax	
			Glass/Flax/Basalt	
			Glass/Hemp/Basalt	
			Glass/Jute	
			Glass/Jute/Vetiver	
			Glass/Kenaf	
			Glass/Kenaf/Bamboo	
			Glass/Palmyra	
			Glass/Pineapple	
			Glass/Sisal	
			Glass/Vetiver	

**Table A3:** Resin and lay-up configuration (*n* denotes the number of data).

Composite Laminate	Resin Type	*n*	Lay-Up	*n*
Hybrid	Thermoset	178	Sandwich	72
	Thermoplastic	13	Intercalated	75
			Not given	42
Non-hybrid	Thermoset	57		
	Thermoplastic	8		

**Table A4:** List of energy absorption mechanisms of hybrid composites.

Label	Hybrid Composite	Radial Crack	Matrix Cracking	Fiber Breakage	Fiber Splitting	Delamination	Indentation	Transverse Crack	Cross-Shaped Crack
SS	Glass/Carbon/Aramid			[44]		[44]	[44]		
SN	Carbon/Flax		[120]	[120]	[120]	[120]	[120]		
SN	Carbon/Flax			[224]		[224]			
SN	Glass/Kenaf			[182]	[182]				
SN	Carbon/Basalt		[127]	[127]		[127]			
SN	Glass/Sisal				[187]				
NN	Basalt/Flax		[162]		[162]				
NN	Jute/Hemp/Flax		[225]	[225]					
NN	Oil palm/Jute		[206]	[206]					
NN	Jute/Cotton		[224]						
SN	Glass/Empty Fruit Bunch		[165]	[165]					
SN	Glass/Jute		[81]	[81]		[81]			
SN	Glass/Jute		[80]	[80]	[80]	[80]			
SN	Glass/Kenaf			[226]					
SN	Carbon/Basalt		[220]			[220]			
SN	Glass/Carbon/ Prosopis juliflora bark fiber			[227]					
SN	Carbon/Flax			[142]		[142]	[142]		[142]
SS	Glass/Carbon	[152]	[152]	[152]		[152]			
SS	Glass/Carbon		[84]	[84]		[84]			
SS	Glass/Carbon			[228]	[228]	[228]	[228]		[228]
SS	Glass/Carbon					[130]			
SS	Carbon/Aramid				[215]	[215]		[215]	
SS	Glass/Carbon		[163]	[163]		[163]			
SS	Glass/Carbon			[229]		[229]			[229]
SN	Carbon/Basalt			[229]		[229]			[229]
SN	Glass/Carbon/Basalt			[229]		[229]			[229]
SS	Glass/Aramid		[183]	[183]		[183]			
SS	Carbon/Aramid		[217]	[217]					
SS	Glass/Carbon		[217]	[217]					
SS	Carbon/Aramid			[219]		[219]			
SS	Glass/Carbon		[79]	[79]		[79]			
SN	Glass/Banana/Sisal		[200]	[200]					
SN	Aramid/Basalt			[230]		[230]	[230]		[230]
NN	Jute/Basalt		[206]			[206]			
SN	Glass/Kenaf			[185]		[185]			
SN	Glass/Banana		[205]			[205]			
SN	Glass/Jute		[128]	[128]		[128]			
SN	Glass/Kenaf		[128]	[128]		[128]			
SN	Carbon/Jute			[231]		[231]			
NN	Flax/Basalt		[150]	[150]		[150]			
SN	Aramid/Basalt		[232]	[232]		[232]			

**Table A5:** List of energy absorption mechanisms of hybrid composites (cntd.)

Label	Hybrid Composite	Penetration	Interfacial Debonding	Bending Cracks	Fiber Pull-Out	Hybrid- Interface Debonding	Fiber Bending	Permanent Deformation	Compression Buckling
SS	Glass/Carbon/Aramid								
SN	Carbon/Flax			[120]					
SN	Carbon/Flax								
SN	Glass/Kenaf		[182]		[182]				
SN	Carbon/Basalt	[127]			[127]	[127]			
SN	Glass/Sisal				[187]				
NN	Basalt/Flax								
NN	Jute/Hemp/Flax		[225]		[225]				
NN	Oil palm/Jute		[206]		[206]				
NN	Jute/Cotton		[224]		[224]				
SN	Glass/Empty Fruit Bunch				[165]				
SN	Glass/Jute	[81]							
SN	Glass/Jute		[80]		[80]				
SN	Glass/Kenaf								
SN	Carbon/Basalt				[220]				
SN	Glass/Carbon/ Prosopis juliflora bark fiber					[227]			
SN	Carbon/Flax	[142]	[142]		[142]				
SS	Glass/Carbon	[152]			[152]				
SS	Glass/Carbon								
SS	Glass/Carbon							[228]	
SS	Glass/Carbon					[130]			
SS	Carbon/Aramid	[215]	[215]						[215]
SS	Glass/Carbon		[163]		[163]				
SS	Glass/Carbon	[229]						[229]	
SN	Carbon/Basalt	[229]						[229]	
SN	Glass/Carbon/Basalt	[229]						[229]	
SS	Glass/Aramid				[183]				
SS	Carbon/Aramid				[217]				
SS	Glass/Carbon		[217]		[217]				
SS	Carbon/Aramid								
SS	Glass/Carbon							[79]	
SN	Glass/Banana/Sisal								
SN	Aramid/Basalt							[230]	
NN	Jute/Basalt								
SN	Glass/Kenaf		[185]		[185]				
SN	Glass/Banana				[205]				
SN	Glass/Jute	[128]							
SN	Glass/Kenaf								
SN	Carbon/Jute								
NN	Flax/Basalt				[150]				
SN	Aramid/Basalt				[232]				
